# Mathematical Model for Evaluation of Tumor Response in Targeted Radionuclide Therapy with ^211^At Using Implanted Mouse Tumor

**DOI:** 10.3390/ijms232415966

**Published:** 2022-12-15

**Authors:** Yoshiharu Yonekura, Hiroshi Toki, Tadashi Watabe, Kazuko Kaneda-Nakashima, Yoshifumi Shirakami, Kazuhiro Ooe, Atsushi Toyoshima, Hiroo Nakajima, Noriyuki Tomiyama, Masako Bando

**Affiliations:** 1 Institute for Radiation Sciences, Osaka University, Suita 565-0871, Japan; 2 Research Center for Nuclear Physics, Osaka University, Suita 565-0047, Japan; 3 Health Care Division, Health and Counseling Center, Osaka University, Toyonaka 560-0043, Japan; 4 Department of Nuclear Medicine and Tracer Kinetics, Osaka University Graduate School of Medicine, Suita 565-0871, Japan; 5 Department of Radiology, Osaka University Graduate School of Medicine, Suita 565-0871, Japan; 6 Yukawa Institute for Theoretical Physics, Kyoto University, Kyoto 606-8502, Japan

**Keywords:** targeted radionuclide therapy, mathematical model, alpha therapy, ^211^At

## Abstract

Recent introduction of alpha-emitting radionuclides in targeted radionuclide therapy has stimulated the development of new radiopharmaceuticals. Preclinical evaluation using an animal experiment with an implanted tumor model is frequently used to examine the efficiency of the treatment method and to predict the treatment response before clinical trials. Here, we propose a mathematical model for evaluation of the tumor response in an implanted tumor model and apply it to the data obtained from the previous experiment of ^211^At treatment in a thyroid cancer mouse model. The proposed model is based on the set of differential equations, describing the kinetics of radiopharmaceuticals, the tumor growth, and the treatment response. First, the tumor growth rate was estimated from the control data without injection of ^211^At. The kinetic behavior of the injected radionuclide was used to estimate the radiation dose profile to the target tumor, which can suppress the tumor growth in a dose-dependent manner. An additional two factors, including the time delay for the reduction of tumor volume and the impaired recovery of tumor regrowth after the treatment, were needed to simulate the temporal changes of tumor size after treatment. Finally, the parameters obtained from the simulated tumor growth curve were able to predict the tumor response in other experimental settings. The model can provide valuable information for planning the administration dose of radiopharmaceuticals in clinical trials, especially to determine the starting dose at which efficacy can be expected with a sufficient safety margin.

## 1. Introduction

Targeted radionuclide therapy (TRT) is an attractive approach to cure patients with intractable cancer. Recent introduction of alpha-emitting radionuclides in TRT has encouraged the medical community to develop new treatment methods with a variety of radioactive compounds [1,2]. To examine the efficacy and the safety of the treatment method, an animal experiment with an implanted tumor model is frequently used as a preclinical evaluation. The successful treatment can suppress tumor growth, and the information obtained from the multiple experiments with various doses of the administered drug is used for the planning of the clinical trial. The experimental design of the radionuclide treatment is usually determined by the expected radiation doses in the tumor and the normal tissues after the administration of the radiolabeled compound that is to be examined. 

The biological effects of radiation have been studied extensively for external beam radiotherapy [3,4,5,6]. Treatment protocols have been established based on the precise dosimetry and the predicted response in tumors and surrounding healthy tissues. In radionuclide therapy, however, such an approach has not been possible, and the treatment is often repeated until complete recovery is reached. As TRT is expected to play an important role in curing the patient, a more quantitative approach is necessary for treatment planning and also for the assessment of treatment response.

For this purpose, we propose a mathematical model for evaluation of tumor response in TRT, considering the kinetics of radiopharmaceuticals, tumor growth, and treatment response. As these factors involve temporal changes, the use of differential equations is a unique approach to describe the behavior of each process. We applied the model to the data obtained from the previous animal experiment with ^211^At treatment in a thyroid cancer mouse model [7].

## 2. Results

### 2.1. Modeling of the Drug Delivery

**Figure 1:**
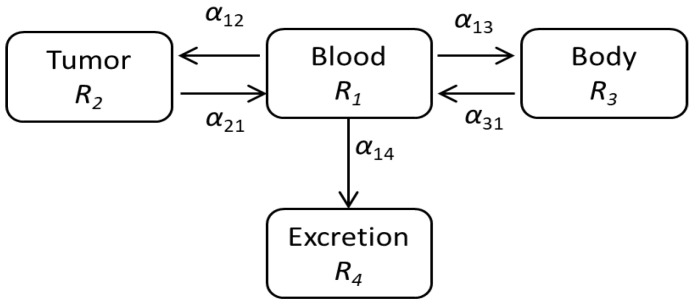
The drug delivery model describing the behavior of ^211^At in mouse. The radiating material ^211^At is delivered to the tumor and other parts of the body through blood and eventually is excreted from body.

To simulate the tumor response by TRT, we first estimated the temporal changes in radioactivity in the tumor after the intravenous injection of ^211^At. Regarding the delivery of ^211^At in mice, we considered a model consisting of four boxes, as shown in Figure 1: blood, tumor, body organs, and excretion. We expressed the drug delivery model in the coupled differential equations, where the temporal changes of *R*’s were calculated using the parameters α’s (see details in Materials and Methods).

**Figure 2:**
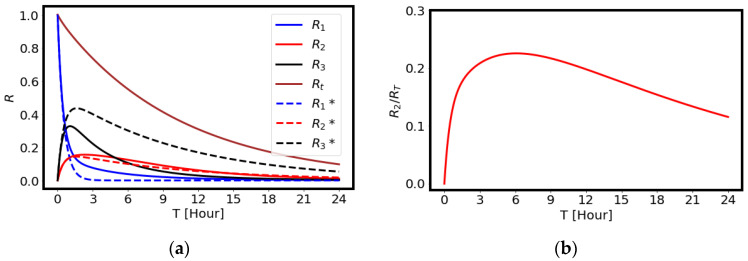
The calculated results of radioactivity in each box. (**a**) The amounts of radiating materials in blood (*R*_1_), tumor (*R*_2_), body (*R*_3_), and total activity (*R_T_*) for the case where the initial amount is set to *R*_1_ = 1. The solid curves are calculated rigorously, including the return paths, while the dashed curves are those of the analytical expressions without considering the return paths. (**b**) The amount of the radiating material in the tumor considering the decay rate *y* is shown as a function of time.

Figure 2 shows the calculated amounts of ^211^At in the tumor, blood, and body organs. The amount obtained by rigorous calculation considering all parameters are denoted by the solid curves, and those without consideration of the return paths, $\alpha_{21}$ and $\alpha_{31}$, are denoted by the dashed curves in the left figure. It is possible to express the solutions analytically once the return paths are dropped in the drug delivery model. The radiation dose in the tumor shows a maximum value around three hours after the injection, as shown by the red solid curve, while ^211^At is cleared from the blood rapidly, as shown by the black solid curve. The calculated results suggest that most of the radiation dose in the tumor is completed within a day due to the short physical half-life (7.21 h) of ^211^At. The effects of the return terms are calculated by setting $\alpha_{21}=\alpha_{31}=0$, which are shown by the red dashed curve and the black dashed curve. The difference is not large for *R*_2_, since the return coefficient $\alpha_{21}$ is small in our parameter choice. Hence, for the estimate of the radiation effect in the following simulation of the cancer treatment, we employed the analytical expression for cancer therapy.

Shown in the right of Figure 2 is the ratio of the radiating material in the tumor and the decay curve of ^211^At, which is usually provided as the renormalized amount of the radiating material. The renormalized amount is compared well with the data obtained by previous work [7]: 23% at three hours and 12% at 24 h after the administration of ^211^At.

### 2.2. The Effects of Radiation on Tumor Volume

**Figure 3:**
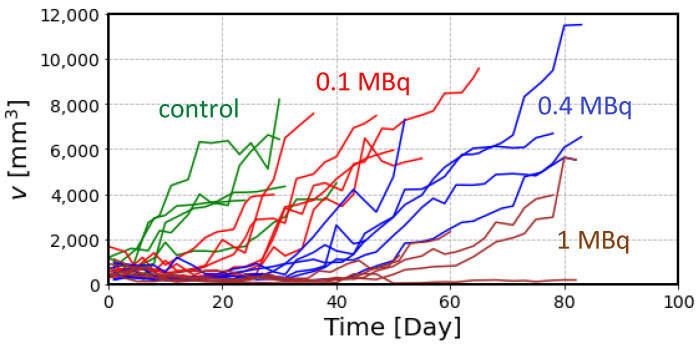
Experimental data of tumor volume in each mouse obtained from the previous experiment with ^211^At treatment [7]. Each polygonal line represents the measured tumor volume in each mouse classified into 4 groups according to the administered dose: control (green), 0.1 MBq (red), 0.4 MBq (blue) and 1 MBq (brown).

The experimental data of tumor size after the treatment with ^211^At are plotted in Figure 3. The large variation shown here is due to the individual variability of tumor growth in each mouse, in addition to the measurement error. Following the administration of ^211^At, most of the tumors showed a gradual decrease in volume but regrowth started in a later phase, except in one mouse which received 1 MBq of ^211^At.

We first estimated the tumor growth rate, *λ*, from the control mice data up to 10 days. Table 1 shows the results of the regression analysis of the tumor growth data in control mice. In spite of the large variation in the initial tumor volume, *V*_0_, the proliferation rate of the tumor, *λ*, showed less variation, and we used the value of 0.14/day as the tumor growth rate for the following analysis to examine the effects of radiation.

**Figure 4:**
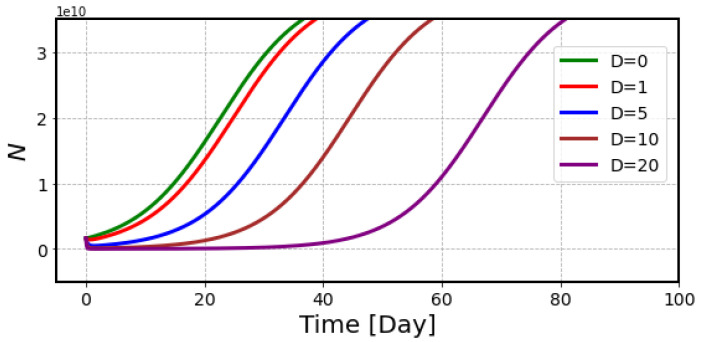
Simulation curves of the number of tumor cells after treatment with ^211^At with doses from 1 Gy to 20 Gy. We used *λ* = 0.14/day, *N*_0_ = 2 × 10^9^, and *N_m_* = 4 × 10^10^.

Figure 4 illustrates the number of tumor cells with ^211^At treatment, which decreases quickly after receiving the radiation but gradually recovers in the later period. It is seen that the recovery is delayed in the high-dose curve, but the slope in the regrowth phase is exactly same as for control data. The data show that the number of survived cells is determined by radiation dose, and these cells go back to the proliferation phase thereafter.

**Figure 5:**
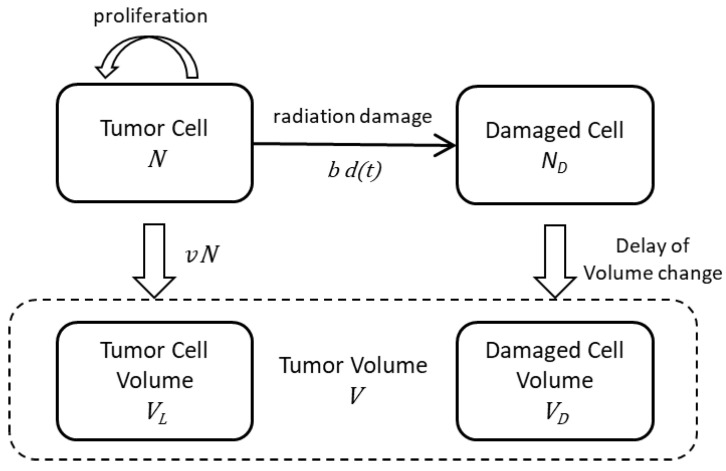
The schematic view of the proposed model. We assume that the cancer volume consists of the viable tumor cell volume, *V_L_*, and the damaged cell volume, *V_D_*, which are related with the corresponding numbers, *N* and *N_D_*. The tumor cells proliferate with the growth rate, *λ,* modified slightly by the damage of cancer tissue due to radiation. The cell damage is caused by the radiation with the radiation dose, *d*(*t*).

Comparison of these simulation curves with the experimental data of the tumor volume shown in Figure 3 suggested that it is necessary to include additional parameters to reproduce the experimental data. Two important factors should be considered: (i) the gradual decrease in tumor volume after the radiation treatment and (ii) a slower slope during the recovery phase after the treatment, particularly for high radiation doses. Considering these factors, we propose a model to simulate the volume change of tumor cells by radiation, as shown in Figure 5.

Another problem is the recovery of tumor growth after the treatment. The experimental data in Figure 3 show that the slope of tumor regrowth is slower, particularly in the case of high-dose treatment, than in the control mice, suggesting an effect on tumor growth rate after radiation. We assumed that tumor growth is impaired by the total dose received by that time and introduced *λ_mod_* as a factor to modify the tumor growth rate by radiation.

**Figure 6:**
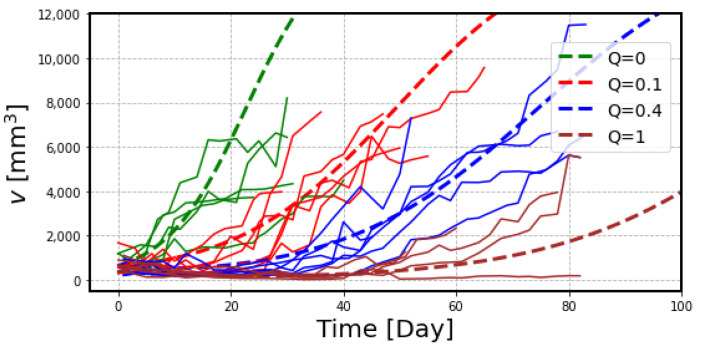
Estimated dose-response curves to reproduce the experimental data of ^211^At treatment in xenograft model mice. Dashed curve lines demonstrate the tumor growth curves by administration of 0.1 MBq (*Q* = 0.1), 0.4 MBq (*Q* = 0.4), and 1 MBq (*Q* = 1) in comparison with control mice (*Q* = 0).

Considering these parameters, the results of the estimated tumor volume curves are shown as dashed lines in Figure 6. A slower recovery slope after receiving the high-dose radiation is well reproduced in this figure. In this final process, we used the estimated radiation dose in the tumor, 9.7 Gy, from the previous work [7] for administration of 1 MBq ^211^At. In spite of a large variation in the experimental data, the simulated curves can reproduce the data.

## 3. Discussion

The animal experiment is the most important preclinical process before the clinical trials of TRT. The obtained results are directly linked to the design of the clinical trials. Mathematical models can provide important information to overcome the limited animal data. In this study, we proposed a model to simulate the volume changes of implanted tumors after the administration of ^211^At, but it can be applied to any animal experiment of TRT. This model is based on three key parameters: (i) the tumor growth rate without radiation, (ii) the effect of radiation to decrease the tumor volume, and (iii) the factor to modify the tumor regrowth rate after radiation. In order to estimate these parameters, we needed the data sets of temporal changes of tumor volume with or without radiation and the radiation profile in the tumor following the administration of radiopharmaceuticals.

**Figure 7:**
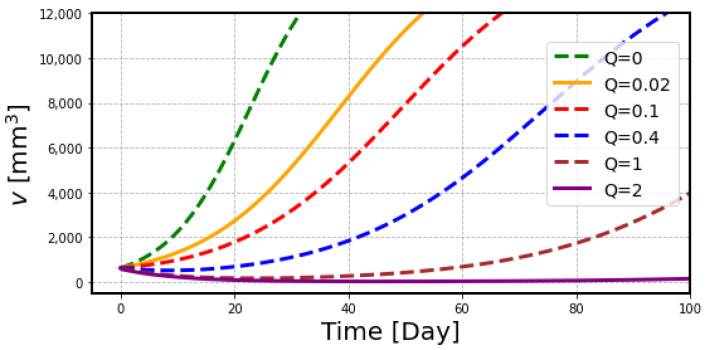
Calculated dose-response curves to predict the tumor response to ^211^At treatment. Two conditions, 0.02 MBq (*Q* = 0.02) in yellow and 2 MBq (*Q* = 2) in purple, are shown as additional curve lines for the results in Figure 6.

One of the great advantages of the mathematical model is to reproduce the animal experiment with different treatment protocols. The data of the experiment are often limited due to the complicated experimental setup and limited resources. For example, Figure 7 shows the simulation curves calculated for the administration doses of ^211^At beyond the range of the experimental data. The model can provide valuable information for planning the administration dose of radiopharmaceuticals in clinical trials, especially to determine the starting dose at which efficacy can be expected with a sufficient safety margin.

TRT usually requires multiple treatment doses to cure a patient with intractable malignant tumors. The treatment protocols are often based on the previous experience. The EU has required the individual treatment planning for all radiotherapeutic procedures, including radionuclide therapy [8]. ICRP also recommended improving the dosimetry for the individual dose estimate in radionuclide therapy [9]. This is particularly important for the use of alpha-emitting radionuclides with high linear energy transfer (LET) radiation. High LET radiation, including alpha particles, induces double-strand breaks (DSBs) in DNA and can provide an effective treatment of tumors. However, it may also cause severe damage to the healthy tissue cells, which should be considered in clinical treatment planning. The proposed model can be used to estimate the effects of repeated administration of radiopharmaceuticals, assuming a similar radiation profile to the first treatment or a modified dose in the subsequent repeated treatment. This treatment approach with multiple low-dose administrations is beneficial to the patient, as it minimizes the damage to the healthy tissues, but needs to be optimized in the treatment protocol, considering the control of tumor regrowth.

The results obtained in this study provide various suggestions for understanding tumor growth and the effects of radiation. Suppression of the tumor regrowth rate after ^211^At treatment can be explained by alterations to the characteristics of tumor cells or tissue environment by radiation. It has been shown that rapidly growing tumor cells are more radiosensitive than slowly growing tumor cells [3]. Therefore, it is possible that the residual tumor cells, which survived after radiation treatment, may grow slowly. Cancer stem cells, which have been reported to be radioresistant, may play a role in this process [10]. We should also consider the possible effects on the immune system after the treatment [11].

The model used in this study needs to be improved by being applied to other treatment protocols and tumor models. The parameters used in this study were adjusted to reproduce the previous experiment with [^211^At]NaAt treatment in K1-NIS xenograft model mice. In this study, we used the *b* value, which is responsible for decreasing the number of tumor cells, of 0.3/Gy. A similar simulation study revealed lower values for external beam radiotherapy [12], supporting the efficient treatment effects of ^211^At [13,14,15]. The therapeutic effects of ^211^At could be predicted by relative biological effectiveness (RBE), but the evaluation of RBE for radionuclide therapy is complicated and requires careful consideration of the methods used [16]. Therefore, we expect that the use of the proposed model can be used to estimate the RBE value of radionuclide treatment in vivo.

In addition to RBE, we may want to consider the effects of dose rate in TRT. The tumor cells received most of the radiation dose within a day after the administration of ^211^At due to the relatively short physical half-life (7.21 h). It may be interesting to compare the results with other alpha-emitting radionuclides of a longer physical half-life, such as ^225^Ac. It is conceivable that TRT may require the best treatment protocol, considering the kinetic behavior, RBE, and dose rate of the radionuclide used, and the present model can be used to compare the results of different treatment methods.

## 4. Materials and Methods

### 4.1. Animal Experiment

The data obtained from the previous animal experiment of ^211^At treatment in a mouse xenograft model were reanalyzed in this study [7]. K1 cells (human papillary thyroid carcinoma) expressing the sodium/iodide symporter (NIS) gene were subcutaneously injected into the SCID mice (1–2 × 10^7^ cells). The mice were treated by intravenous administration of ^211^At solution (control, 0.1 MBq, 0.4 MBq, 1 MBq) when the tumor size reached approximately 10 mm in diameter (37 days on average). The tumor size was measured by an external caliper, and the tumor volume was calculated using the assumption of an oblate ellipsoid.

### 4.2. Estimation of the Drug Delivery

In order to analyze the drug delivery model expressed by the box model shown in Figure 1, we wrote differential equations for the numbers of radionuclides, *R*, in these boxes: *R*_1_ for blood, *R*_2_ for tumor, *R*_3_ for body organs, and *R*_4_ for excretion.

(1) $$\frac{dR_{1}\left(t\right)}{dt}=-\alpha_{12}R_{1}-\alpha_{13}R_{1}-\alpha_{14}R_{1}+\alpha_{21}R_{2}+\alpha_{31}R_{3}-yR_{1}$$

$$\frac{dR_{2}\left(t\right)}{dt}=\alpha_{12}R_{1}-\alpha_{21}R_{2}-yR_{2}$$

$$\frac{dR_{3}\left(t\right)}{dt}=\alpha_{13}R_{1}-\alpha_{31}R_{3}-yR_{3}$$

$$\frac{dR_{4}\left(t\right)}{dt}=\alpha_{14}R_{1}-yR_{4}$$

Here, *y* denotes the decay rate of the radiating material. The coefficients *α* are fixed so as to reproduce the experimental behavior. The total number of radionuclides, *R_T_* (=*R*_1_ + *R*_2_ + *R*_3_ + *R*_4_), decreases with the decay rate, *y*:

(2) $$\frac{dR_{T}}{dt}=-yR_{T}\left(t\right),\;\mathrm{with}\;R_{T}=R_{1}\left(t=0\right)e^{-yt}$$

In the case where the coefficients of the return paths are zero, $\alpha_{21}=0$ and $\alpha_{31}=0$, we can solve the differential equations analytically. The solutions are

(3) $$R_{1}=R_{1}\left(t=0\right)e^{-At}$$

and

(4) $$R_{i}=R_{1}\left(t=0\right)\frac{\alpha_{1i}}{B}(1-e^{-Bt})e^{-yt}$$

with *i* = 2, 3, 4. Here, $A=\alpha_{12}+\alpha_{13}+\alpha_{14}+y$, and $B=\alpha_{12}+\alpha_{13}+\alpha_{14}$. This equation indicates that *R*_2_ increases linearly with the slope $\alpha_{12}$ and has a peak and decreases with the decay rate y. The amount *R*_2_ is determined by the ratio of $\alpha_{12}/B$. The initial condition can be provided as follows: the injected activity, *Q*, for *R*_1_ (t = 0), and 0 for other parameters

*R*_1_ (*t* = 0) = *Q*, *R*_2_ (t = 0) = 0, *R*_3_ (t = 0) = 0, *R*_4_ (t = 0) = 0. (5)

These equations provide the following numerical results for the radiation dose profile by taking the parameters:

(6) $$\alpha_{12}=0.33,\;\;\alpha_{13}=1,\;\;\alpha_{14}=0.5,\;\;\alpha_{21}=0.1,\;\;\alpha_{31}=0.5,\;\;\;y=log\left(2\right)/7.21=0.096.$$

Next, we wanted to estimate the amount of radiation in the tumor using the analytical expression for *R*_2_ by integrating *R*_2_(*t*) over time:

(7) $$\int_{0}^{\infty }R_{2}\left(t\right)\;dt=Q\frac{\alpha_{12}}{B}\left(\frac{1}{y}-\frac{1}{A}\right)=Q\frac{\alpha_{12}}{yA}$$

This expression denotes the total amount of radiating material in the tumor, where one ^211^At emits two alpha particles of energy 5.9 and 7.5 MeV. Since the becquerel (Bq) is the unit for the number of emitting particles per second, we should take an average of the two energies per one emission. The radiation dose of 1 MBq of ^211^At in a tumor with the weight 1.5 g provides 5.45 Gy per hour, so the total dose in the tumor becomes 9.7 Gy using the above Equation (7) with the parameters (6), corresponding to the calculated value of Watabe et al. [7]. Based on this observation, we calculated the radiation dose rate for each administration dose of ^211^At.

### 4.3. Estimation of Radiation Effects on Tumor Volume

The treatment model for cancer therapy by external radiation using X-rays has been studied by Bando et al. [12], and we applied a similar concept to TRT. We assume that the implanted tumor cells grow continuously with a certain limit on the volume. We start with a simple consideration, where the number of the tumor cells, *N*, increases with the growth rate, *λ*, with the maximum number, *N_m_*, and decreases with the killing rate, *b*, due to the radiation with the dose rate, *d*(*t*).

(8) $$\frac{dN}{dt}=\left(\lambda -bd\left(t\right)\right)N\left(1-\frac{N}{N_{m}}\right)$$

Here, we can calculate the radiation dose rate, *d*(*t*), from the amount of radionuclide in the tumor, *R*_2_(*t*), considering the temporal changes in its activity.

In control mice during the growth phase, tumor volume, *V*, is considered to be parallel to the number of cells, *N*, and the tumor growth rate, *λ*, can be estimated from the early stage of tumor growth. Here, we assume the volume of a cancer cell as *v* and *V* = *vN*.

(9) $$\frac{dV}{dt}=\left(\lambda -bd\left(t\right)\right)V\left(1-\frac{V}{V_{m}}\right)$$

If *d*(*t*) = 0, and *V* is small compared to *V_m_*, we get the following simple equation and corresponding analytical solution.

(10) $$\frac{dV}{dt}=\lambda V$$

(11) $$V=V_{0}\exp(\lambda t)$$

We estimated the tumor growth rate, *λ*, from the control mice data up to 10 days using the simple expression (11). 

Numerical analysis of Equation (8) provided the simulation curves for the number of tumor cells after treatment with ^211^At by setting the initial conditions of *N*_0_ as 2 × 10^9^ and *b* as 0.3/Gy. 

The tumor volume slowly decreased after receiving the radiation dose, while the damaged tumor cells quickly lost the ability to proliferate. The gradual decrease in the cancer volume is attributed to the gradual loss of volume of the damaged cells. To reflect this relation, we introduced the time-delay effect for the volume change of damaged tumor cells. Here, we considered two types of tumor cells: those with cancer cell proliferation and damaged cells without the ability to proliferate. The tumor volume is a sum of the volume of proliferating cells, *V_L_*, and that of damaged cells, *V_D_*.

(12) $$V\left(t\right)=V_{L}\left(t\right)+V_{D}\left(t\right)$$

Then we get the following equation to convert the number of cells into their volume, considering the apparent volume size of the tumor cell, *v*, and $V_{L}\left(t\right)=vN(t$), with the single cell volume, *v*.

(13) $$V\left(t\right)=vN\left(t\right)+V_{D}\left(t\right)$$

Proliferating tumor cells, *N*, and damaged cells, *N_D_*, can be described by the following equations, neglecting the saturation volume effect.

(14) $$\frac{dN}{dt}=\left(\lambda -bd\right)N$$

(15) $$\frac{dN_{D}}{dt}=bdN$$

The delay effect of tumor volume change of damaged cells can be described as a function of time.

(16) $$V_{D}\left(t\right)=vN_{D}e^{-c\left(t-t_{0}\right)}$$

This simple formula is obtained by approximating that the time duration, *t*_0_, (<1 day) of the radiation effect is much smaller than the time 1/*c* (~10 days) of the damaged cell decrease. In the actual calculation, we set *t*_0_ = 0 and *c* = 0.1.

Finally, we introduced *λ_mod_* as a factor to modify the tumor growth rate by radiation, assuming that tumor growth is impaired by the total dose, *D*(*t*), received by that time.

(17) $$\lambda_{mod}\left(t\right)=\frac{\lambda }{1+D{\left(t\right)}^{1/4}}$$

(18) $$D\left(t\right)=\int_{0}^{t}d\left(\tau \right)d\tau$$

This modified tumor growth rate, *λ_mod_*, calculated by Equation (17) was applied to Equations (8) and (9) for the estimation of tumor growth curves with ^211^At treatment.

(19) $$\frac{dN}{dt}=\left(\lambda_{mod}\left(t\right)-bd\left(t\right)\right)N\left(1-\frac{N}{N_{m}}\right)$$

(20) $$\frac{dV}{dt}=\left(\lambda_{mod}\left(t\right)-bd\left(t\right)\right)V\left(1-\frac{V}{V_{m}}\right)$$

## 5. Conclusions

The present study demonstrated the value of a mathematical model for the evaluation of the treatment efficiency of TRT in an implanted mouse tumor model. The model can be used to predict the treatment response and to plan the administration dose of radiopharmaceuticals in clinical trials, especially to determine the starting dose at which efficacy can be expected with a sufficient safety margin.

## Figures and Tables

**Table 1:** Results of the regression analysis for tumor growth data in control mice up to 10 days with the number of data points (*n* = 6).

Parameter	Mean	Standard Deviation
*λ* (/day)	0.14	0.08
*V*_0_ (mm^3^)	577	372

## Data Availability

All data are included in this published article.
